# Clinical and Radiographic Outcomes of Different Root Canal Obturation Materials in Primary Teeth: A Retrospective Observational Study

**DOI:** 10.7759/cureus.117451

**Published:** 2026-10-04

**Authors:** Pooja S Sankpal, Arwa Hussain Chaiwala, Sachin Franklin, Harveen Kaur, Sameer Chauhan, D. Rupak Kumar

**Affiliations:** 1 Department of Pediatric and Preventive Dentistry, Bharati Vidyapeeth (Deemed to be University) Dental College and Hospital, Sangli, IND; 2 Department of Dentistry, Southern Illinois University (SIU) School of Family and Community Medicine, Springfield, USA; 3 Department of Dentistry, Central Counties Health Centers, Springfield, USA; 4 Department of Oral Medicine and Radiology, Dr. Harvansh Singh Judge Institute of Dental Sciences and Hospital, Panjab University, Chandigarh, IND; 5 Department of Prosthodontics, K. M. Shah Dental College and Hospital, Sumandeep Vidyapeeth (Deemed to be University), Vadodara, IND; 6 Department of Pedodontics and Preventive Dentistry, Priyadarshini Dental College and Hospital, Pandur, IND

**Keywords:** dental materials, primary teeth, pulpectomy, root canal filling materials, treatment outcome

## Abstract

Introduction: Pulpectomy is commonly performed to retain the primary teeth affected by irreversible pulpal disease or necrosis. The choice of root canal filling material may influence treatment outcomes, although the comparative clinical evidence remains inconsistent. This study aimed to compare the clinical and radiographic outcomes of three root canal filling materials used for pulpectomy of primary molars.

Materials and methods: This retrospective observational study reviewed the records of children who underwent pulpectomy of the primary molars. Forty-five eligible cases of pulpectomy (per patient, one primary molar tooth) were included and categorized into three groups of 15, according to the material used: zinc oxide eugenol (ZOE), Metapex, and Endoflas. Clinical and radiographic outcomes were assessed at 12 months of follow-up. The obturation quality and adverse radiographic findings were also evaluated. Statistical analysis was performed using the chi-square test, effect-size estimation, and binary logistic regression.

Results: Clinical success was observed in 10 (66.7%), 13 (86.7%), and 14 (93.3%) teeth in the ZOE, Metapex, and Endoflas groups, respectively (p = 0.013). Radiographic success was observed in nine (60.0%), 12 (80.0%), and 13 (86.7%) teeth, respectively (p = 0.044). Overall success was achieved in eight (53.3%), 11 (73.3%), and 12 (80.0%) teeth, respectively (p = 0.030). Optimal obturation was more frequent with Metapex and Endoflas than ZOE (p = 0.025). Pathological root resorption and persistent/new radiolucency were also significantly more frequent with ZOE treatment. In the regression analysis, Metapex and Endoflas were associated with greater odds of overall success than ZOE.

Conclusion: Within the limitations of this retrospective study, Metapex and Endoflas were associated with more favorable clinical and radiographic outcomes than ZOE. Further prospective studies are required to confirm these findings.

## Introduction

Pulpectomy is a widely accepted treatment modality for preserving the primary teeth affected by irreversible pulpitis or pulpal necrosis until normal exfoliation [1]. Maintaining primary teeth is essential for preserving arch length, masticatory efficiency, speech development, esthetics, and proper eruption of the permanent dentition [2]. The long-term success of pulpectomy depends on several factors, including adequate canal debridement, disinfection, and the use of an obturation material that can effectively seal the root canal system while being compatible with the physiological resorption process of primary teeth [3].

An ideal obturation material for primary teeth should possess antibacterial properties, provide an effective seal against microbial leakage, be non-irritating to periapical tissues, resorb at a rate similar to that of the primary tooth roots, and not interfere with the eruption of the underlying permanent successor [4]. Over the years, several materials have been used for obturation of primary teeth, among which zinc oxide eugenol (ZOE), calcium hydroxide-iodoform paste, and calcium hydroxide-iodoform-silicone paste are the most commonly employed materials [5]. Each material exhibits distinct biological and clinical characteristics that may influence treatment outcomes.

ZOE has traditionally been considered the gold standard because of its antimicrobial properties and handling characteristics; however, concerns have been raised regarding its slow resorption when extruded beyond the apex [5-7]. Calcium hydroxide-iodoform pastes have gained popularity because of their antibacterial activity and favorable resorption profiles [5,8]. Similarly, calcium hydroxide-iodoform-silicone-based materials have been advocated for their ease of placement, biocompatibility, and potential for improved clinical performance [9]. Despite widespread clinical use, the available evidence comparing the long-term clinical and radiographic outcomes of these materials remains inconsistent [5,9].

Therefore, the present study aimed to compare the clinical and radiographic outcomes of different obturation materials used in pulpectomy of primary teeth. The specific objectives were to evaluate the overall success rates associated with ZOE, calcium hydroxide-iodoform paste, and calcium hydroxide-iodoform-silicone paste; assess clinical and radiographic success separately; compare the quality of obturation achieved with each material; and determine the occurrence of pathological root resorption and periapical or furcation radiolucencies during follow-up.

## Materials and methods

Study design and setting

This retrospective observational study was conducted at the Department of Pediatric and Preventive Dentistry (Pedodontics), Bharati Vidyapeeth (Deemed to be University) Dental College and Hospital, Sangli, India. The study was performed through retrospective evaluation of the clinical and radiographic records of children who had undergone pulpectomy of primary molars between January 2016 and February 2021. The treatment was completed before the initiation of the present investigation, and the choice of filling material was made by the treating clinician according to routine clinical practice rather than by the investigators. Considering the retrospective nature of the study and the use of existing archival data, the study was approved and granted a waiver of ethical clearance by the Institutional Ethics Committee. No additional clinical or radiographic procedures were performed for the purpose of the study, and the confidentiality of patient information was maintained throughout the study.

Study population and eligibility criteria

The study population consisted of children aged 5-10 years who had undergone pulpectomy in the primary molars during the predefined record review period. Patient records were identified consecutively from departmental clinical records and screened according to the predetermined eligibility criteria. Records were eligible when they documented pulpectomy treatment in a primary molar by a trained pedodontist with more than five years of experience, complete preoperative clinical information, an interpretable preoperative radiograph, use of one of the three study filling materials, an immediate postoperative radiograph suitable for assessment of obturation quality, and at least six months of postoperative clinical and radiographic follow-up. Records were excluded when clinical documentation was incomplete, radiographs were of poor quality or non-diagnostic, the treated tooth had a developmental anomaly or previous endodontic treatment, a systemic condition potentially affecting healing was documented, or adequate postoperative follow-up was unavailable. The final study cohort comprised 45 treated primary molars (one treated primary molar per patient to maintain independent samples), with 15 teeth allocated to each material group. The eligibility criteria were prespecified and applied consistently during the retrospective record review, in accordance with recommendations for transparent reporting of observational studies [10].

Clinical treatment and classification of study groups

Pulpectomy procedures were performed as part of routine clinical care before this retrospective investigation. For each eligible record, the documented treatment sequence was reviewed, including the preoperative diagnosis, local anesthesia, isolation, access preparation, pulp extirpation, working-length determination, canal preparation, irrigation, canal drying, root canal filling, and definitive coronal restoration, when these details were available in the clinical record. As the study was retrospective, no treatment procedure or material was altered or standardized retrospectively.

Treated teeth were categorized according to the filling material documented in the original clinical records. Group I comprised teeth treated with ZOE (Septodont, Saint-Maur-des-Fossés, France). Group II comprised teeth treated with Metapex, a calcium hydroxide-iodoform root canal filling paste (Meta Biomed Co., Ltd., Cheongju-si, Chungcheongbuk-do, Republic of Korea). Group III comprised teeth treated with Endoflas FS (Laboratorios Sanlor S.A.S., Cali, Colombia). The commercial material documented in the original record was retained for group classification, and no attempt was made to substitute or reclassify the material during the retrospective analysis (Figure 1).

**Figure 1:**
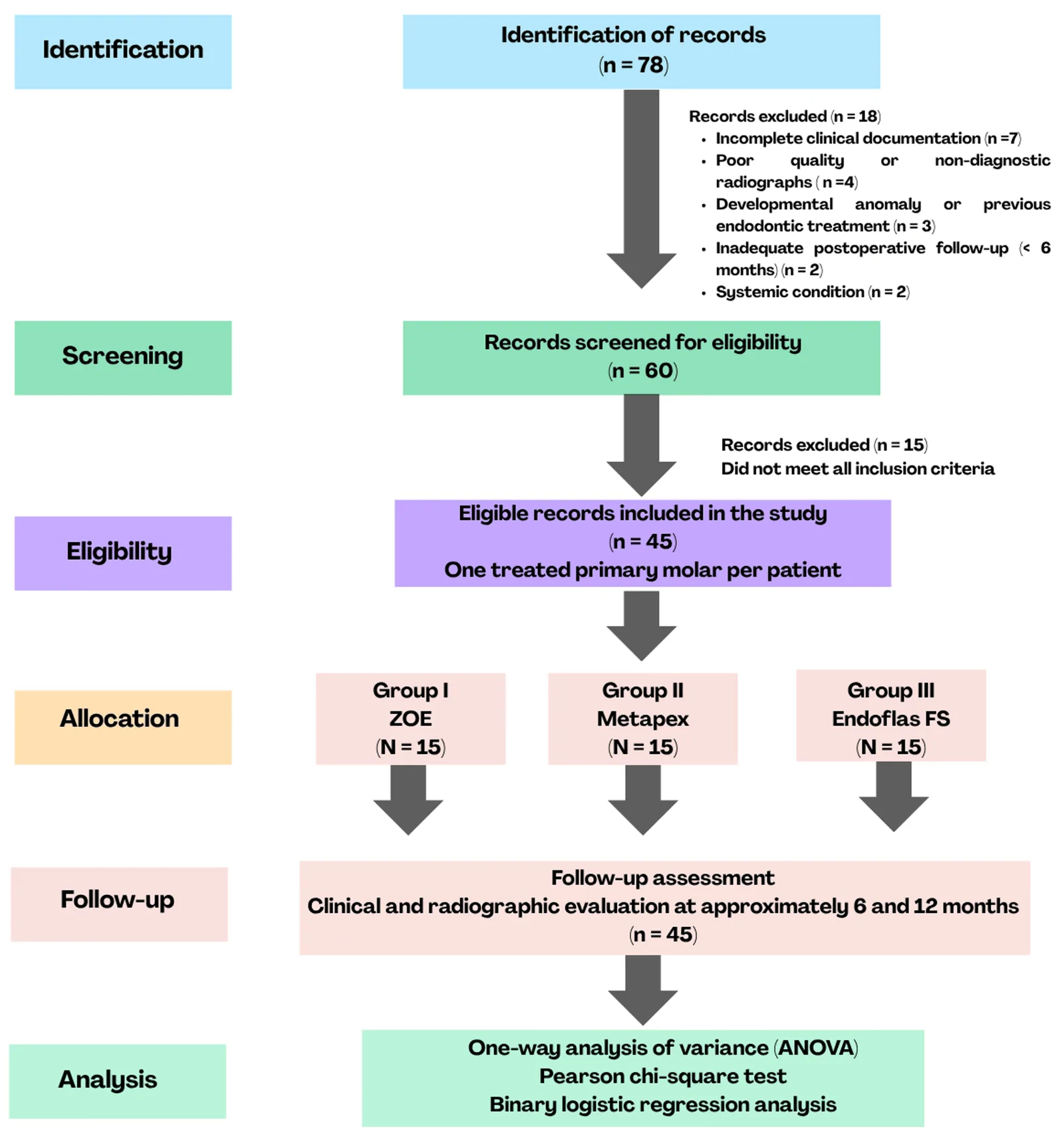
Study flowchart. ZOE: zinc oxide eugenol The image was created by the authors using Canva (Canva Pty Ltd, Sydney, Australia).

Data collection

The demographic variables included age and sex. Clinical variables included tooth type, maxillary or mandibular arch, preoperative diagnosis, and presence or absence of spontaneous pain, swelling, sinus tract, tenderness, and pathological mobility. Tooth type was recorded according to whether the treated tooth was the first or second primary molar. Radiographic variables included the presence or absence of preoperative periapical or furcation radiolucency and other pathological changes. Treatment-related variables included the filling material used and radiographic quality of obturation. Follow-up variables included clinical symptoms and signs, radiographic healing, persistence or development of periapical or furcation radiolucency, pathological internal or external root resorption, and overall treatment outcomes. The follow-up duration was recorded in months.

Radiographic assessment and obturation quality

Preoperative and postoperative radiographs available in clinical records were evaluated for diagnostic quality before inclusion in the analysis. An immediate postoperative radiograph was used to assess the quality of root canal obturation. Filling was categorized as optimal when the material demonstrated a homogeneous appearance and terminated approximately 1-2 mm short of the radiographic apex. An obturation terminating >2 mm short of the radiographic apex was categorized as underfilled, whereas material extending beyond the radiographic apex was categorized as overfilled. These categories were consistently used across all three study groups.

Clinical outcome assessment

Clinical outcomes were assessed based on the findings documented during postoperative follow-up. Clinical success was defined as the absence of all five predefined clinical signs and symptoms: spontaneous pain, swelling, sinus tract, tenderness, and pathological mobility. Each clinical criterion was recorded individually to assess specific postoperative clinical findings. A tooth was considered to have overall clinical success only when all five clinical criteria were simultaneously fulfilled.

Radiographic outcome assessment

Radiographic success was assessed based on the presence or absence of pathological changes on follow-up radiographs. Successful radiographic outcomes required resolution or absence of pre-existing periapical or furcation radiolucency, newly developed radiolucency, and pathological internal or external root resorption. Each component was recorded separately before determining composite radiographic outcomes.

Overall treatment outcome

The primary outcome was the overall clinical and radiographic success at 12 months. Overall success was defined as the simultaneous fulfillment of the predefined clinical and radiographic success criteria. Therefore, a tooth was considered to have overall treatment success only when it was free from all five clinical signs and symptoms and also demonstrated radiographic success, including the absence or resolution of pathological radiographic findings and absence of pathological root resorption. The secondary outcomes included individual clinical criteria, overall radiographic success, obturation quality, pathological root resorption, and persistent or newly developed periapical or furcation radiolucency.

Follow-up assessment

Clinical and radiographic follow-up records were reviewed at approximately six and 12 months after treatment when available. The minimum eligibility requirement was six months of follow-up; however, the principal outcome analysis was based on a 12-month assessment. The follow-up duration was recorded for each eligible tooth.

Examiner calibration and reliability

Clinical and radiographic records were independently evaluated by two calibrated examiners (SF, DRK) using predefined assessment criteria. Before the formal assessment, representative records were jointly reviewed to standardize the interpretation of clinical and radiographic outcome definitions. The examiners were blinded to the clinical outcomes during radiographic assessment wherever feasible. When the filling material could be identified from the radiograph, complete blinding to group allocation was not possible; therefore, predefined objective criteria were consistently applied to minimize observer-related bias.

Inter-examiner agreement was assessed by independently evaluating a subset of records by both examiners, whereas intra-examiner reliability was assessed by repeating the assessment of the same subset after an appropriate interval. Categorical observations were assessed using Cohen’s kappa coefficient, whereas continuous measurements, where applicable, were evaluated using the intraclass correlation coefficient. The intra-examiner agreement was excellent (Cohen’s κ = 0.92), and the inter-examiner agreement was also excellent (Cohen’s κ = 0.90). The reliability assessment was completed before the final statistical analysis.

Sample size calculation

The sample size was calculated a priori using G*Power version 3.1.9.7 (Heinrich Heine University Düsseldorf, Düsseldorf, Germany). The primary outcome was overall clinical and radiographic success at 12 months, and the planned comparison involved three independent groups. Therefore, a chi-square test of independence was selected as the basis for sample size estimation. The effect size was derived from a study by Al-Ostwani et al. [11], who reported 12-month clinical success rates of 87.5% for Endoflas and 75% for Metapex. Based on these data, a medium effect size of w = 0.35 was assumed for the three-group comparison. With an alpha level of 0.05 and statistical power of 80%, the calculated minimum sample size was 42 teeth. To account for approximately 10% potential loss due to incomplete records or inadequate follow-up, the required sample size was increased to 45 teeth, resulting in an equal allocation of 15 teeth per group.

Statistical analysis

Statistical analysis was performed on the coded data by a blinded statistician (SC) using IBM SPSS Statistics for Windows, Version 26 (Released 2018; IBM Corp., Armonk, New York, United States). Continuous variables, including age and follow-up duration, were expressed as mean ± standard deviation and compared among the three study groups using one-way analysis of variance (ANOVA). Eta-squared (η^2^) was reported as the effect size measure for continuous variables. Categorical variables, including sex, arch, preoperative diagnosis, obturation quality, clinical outcomes, radiographic outcomes, pathological root resorption, persistent or newly developed periapical/furcation radiolucency, and overall treatment success, were expressed as frequencies and percentages and compared among the three groups using the Pearson chi-square test. Cramer's V has been reported as an effect size measure for categorical comparisons. For the primary outcome, clinical success, radiographic success, and overall clinical and radiographic success at 12 months were compared among the three groups using the chi-square test. The obturation quality and secondary radiographic outcomes were analyzed using the same approach.

Binary logistic regression analysis was performed to identify factors independently associated with overall treatment success at 12 months. Obturation material was entered as a categorical predictor with ZOE designated as the reference category. The model was adjusted for age, tooth type, arch type, preoperative radiolucency, and obturation quality. Adjusted odds ratios (ORs) and 95% confidence intervals (CIs) were calculated. Model calibration was assessed using the Hosmer-Lemeshow goodness-of-fit test, and Nagelkerke R^2^ was used to describe the explanatory capacity of the model. All statistical tests were two-sided, and statistical significance was set at p < 0.05.

## Results

Baseline characteristics

After screening 78 patients, a total of 45 primary molars were included (one primary molar per patient), with 15 teeth in each study group. The mean age was comparable between the ZOE, Metapex, and Endoflas groups, with no significant difference between the groups (p = 0.691, η^2^ = 0.014). Similarly, no significant differences were observed in the sex distribution, arch, or preoperative diagnosis (all p > 0.05). The follow-up duration was also comparable between the groups (p = 0.892, η^2^ = 0.004), as shown in Table 1.

**Table 1:** Baseline demographic and clinical characteristics of primary molars according to the obturation material used. Data are presented as mean ± standard deviation (SD) for continuous variables and frequencies and percentages for categorical variables. One-way analysis of variance (ANOVA) was used for continuous variables, and the chi-square test (χ^2^) was used for categorical variables. p < 0.05 was considered statistically significant. ZOE: zinc oxide eugenol

Variable		Group I (ZOE) (n = 15)	Group II (Metapex) (n = 15)	Group III (Endoflas) (n = 15)	Test statistic	p-value	Effect size
Age, mean ± SD	Years	6.8 ± 1.9	7.1 ± 1.7	6.5 ± 2.0	F = 0.372	0.691	η^2^ = 0.014
Sex, n (%)	Male	8 (53.3)	7 (46.7)	9 (60.0)	χ^2^ = 0.536	0.765	V = 0.109
	Female	7 (46.7)	8 (53.3)	6 (40.0)			
Arch, n (%)	Maxillary	6 (40.0)	5 (33.3)	7 (46.7)	χ^2^ = 0.712	0.701	V = 0.126
	Mandibular	9 (60.0)	10 (66.7)	8 (53.3)			
Preoperative diagnosis, n (%)	Irreversible pulpitis	9 (60.0)	10 (66.7)	8 (53.3)	χ^2^ = 0.227	0.893	V = 0.071
	Periapical/furcation radiolucency	6 (40.0)	5 (33.3)	7 (46.7)			
Follow-up duration, mean ± SD	Months	12.4 ± 1.1	12.3 ± 1.2	12.5 ± 0.9	F = 0.114	0.892	η^2^ = 0.004

Quality of obturation

Optimal obturation was observed in seven (46.7%), 11 (73.3%), and 12 (80.0%) teeth in the ZOE, Metapex, and Endoflas groups, respectively. Underfilling was more frequent with ZOE, occurring in six (40.0%) teeth, compared to three (20.0%) and two (13.3%) teeth in the Metapex and Endoflas groups, respectively. The overall difference in obturation quality was significant (p = 0.025; Cramer's V = 0.405) (Table 2). Pairwise comparisons demonstrated significantly greater optimal obturation with Endoflas (p = 0.009) and Metapex (p = 0.046) than with ZOE, whereas the difference between Metapex and Endoflas was not significant (p = 0.672).

**Table 2:** Radiographic quality of root canal obturation according to the obturation material used in primary molars. Data are presented as frequencies and percentages. The chi-square test (χ^2^) was used to compare obturation quality among the three groups, and Cramer's V was reported as the effect size. * p < 0.05 was considered statistically significant. ZOE: zinc oxide eugenol

Obturation quality	Group I (ZOE) (n = 15)	Group II (Metapex) (n = 15)	Group III (Endoflas) (n = 15)	Test value (χ^2^)	p-value	Effect size, Cramer’s V
Optimal (1-2 mm short of apex), n (%)	7 (46.7)	11 (73.3)	12 (80.0)	7.39	0.025*	0.405
Underfilled (>2 mm short), n (%)	6 (40.0)	3 (20.0)	2 (13.3)			
Overfilled (beyond apex), n (%)	2 (13.3)	1 (6.7)	1 (6.7)			

Clinical, radiographic, and overall success

Overall, clinical success was observed in 10 (66.7%), 13 (86.7%), and 14 (93.3%) teeth in the ZOE, Metapex, and Endoflas groups, respectively, with a significant between-group difference (χ^2^ = 8.61, p = 0.013; Cramer's V = 0.437). Radiographic success was observed in nine (60.0%), 12 (80.0%), and 13 (86.7%) teeth, respectively (χ^2^ = 6.23, p = 0.044; Cramer's V = 0.372). The primary outcome of overall clinical and radiographic success was achieved in eight (53.3%) teeth in the ZOE group, 11 (73.3%) in the Metapex group, and 12 (80.0%) in the Endoflas group. The differences among the three groups were statistically significant (χ^2^ = 7.03, p = 0.030; Cramer's V = 0.395), as shown in Table 3.

**Table 3:** Clinical, radiographic, and overall treatment success of primary molars at 12-month follow-up according to the obturation material used. Data are presented as frequencies and percentages. The chi-square test (χ^2^) was used to compare clinical, radiographic, and overall treatment success among the three groups, and Cramer's V was reported as the effect size. *p < 0.05 was considered statistically significant. ZOE: zinc oxide eugenol

Outcome	Group I (ZOE) (n = 15)	Group II (Metapex) (n = 15)	Group III (Endoflas) (n = 15)	Test value (χ^2^)	p-value	Effect size, Cramer’s V
Clinical success, n (%)	10 (66.7)	13 (86.7)	14 (93.3)	8.61	0.013*	0.437
Radiographic success, n (%)	9 (60.0)	12 (80.0)	13 (86.7)	6.23	0.044*	0.372
Overall success, n (%)	8 (53.3)	11 (73.3)	12 (80.0)	7.03	0.030*	0.395

The individual clinical findings shown in Figure 2 generally favor Metapex and Endoflas. Overall clinical success, defined as absence of all symptoms, was observed in 10 (66.7%) patients in Group I, 13 (86.7%) in Group II, and 14 (93.3%) in Group III. Absence of pain was recorded in 11 (73.3%), 14 (93.3%), and 14 (93.3%) patients, respectively. Absence of swelling and mobility followed a similar pattern, with the highest proportions generally observed in Group III. Absence of sinus tract was achieved in 13 (86.7%) patients in Group I and all 15 (100%) patients in Groups II and III. Radiographic success was observed in nine (60.0%), 12 (80.0%), and 13 (86.7%) patients in Groups I, II, and III, respectively. Resolution/no new periapical-furcation radiolucency and absence of pathological root resorption also showed higher success proportions in Groups II and III.

**Figure 2:**
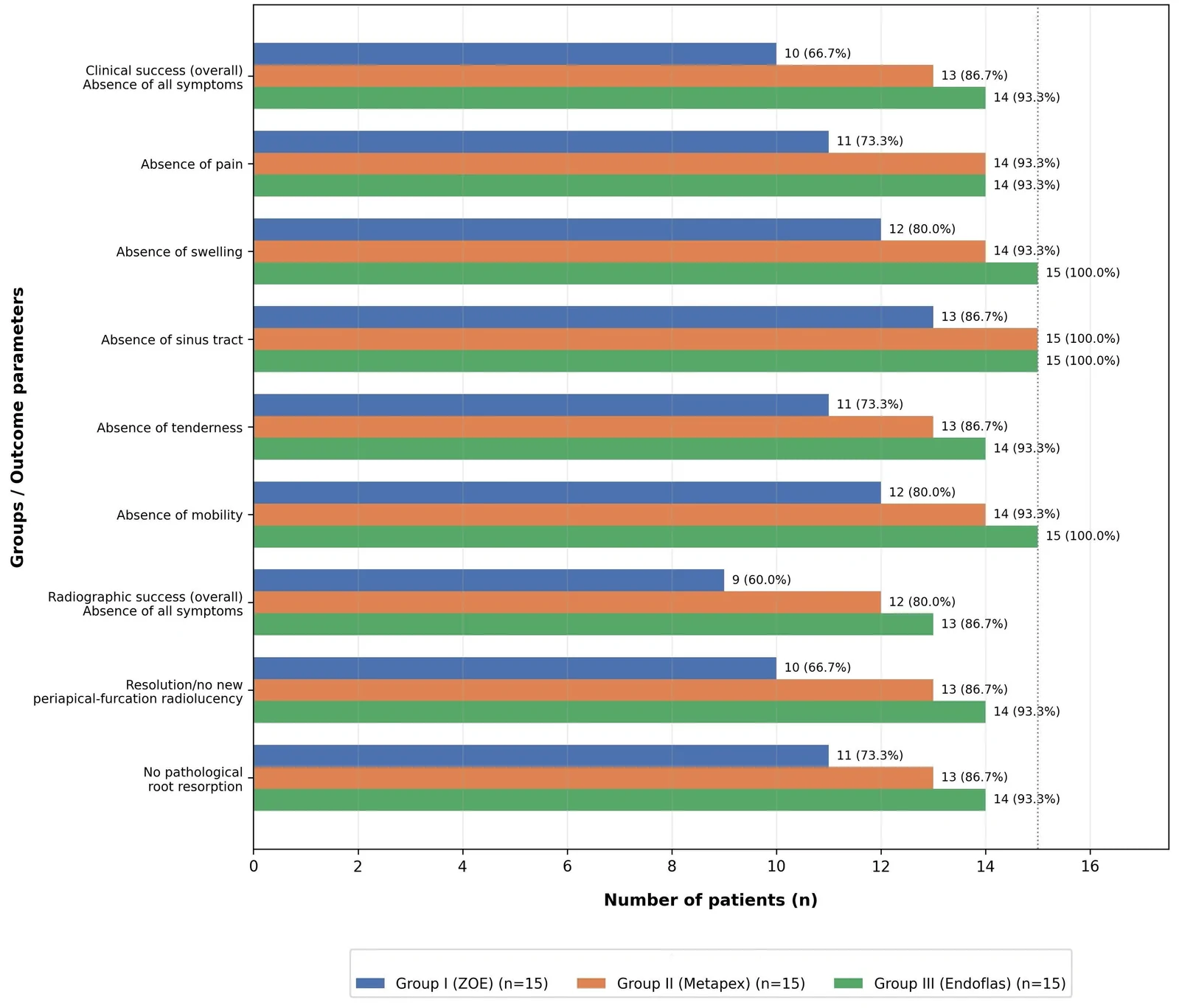
Clinical and radiographic success of primary molars at 12-month follow-up according to the obturation material used. Data are presented as frequencies and percentages. Clinical success represents the absence of all predefined clinical signs and symptoms, whereas radiographic success represents resolution or absence of periapical/furcation radiolucency and absence of pathological root resorption. ZOE: zinc oxide eugenol

Secondary radiographic outcomes

Pathological root resorption occurred in four (26.7%) teeth in the ZOE group compared with one (6.7%) tooth in the Metapex and Endoflas groups, demonstrating a significant between-group difference (χ^2^ = 7.78, p = 0.020; Cramer's V = 0.416). Persistent or newly developed periapical/furcation radiolucency was observed in five (33.3%), two (13.3%), and one (6.7%) tooth in the ZOE, Metapex, and Endoflas groups, respectively. This difference was statistically significant (χ^2^ = 7.03, p = 0.030; Cramer's V = 0.395) (Table 4).

**Table 4:** Pathological root resorption and persistent or newly developed periapical or furcation radiolucency at 12-month follow-up according to the obturation material used. Data are presented as frequencies and percentages. The chi-square test (χ^2^) was used to compare clinical, radiographic, and overall treatment success among the three groups, and Cramer's V was reported as the effect size. *p < 0.05 was considered statistically significant. ZOE: zinc oxide eugenol

Secondary outcome	Group I (ZOE) (n = 15)	Group II (Metapex) (n = 15)	Group III (Endoflas) (n = 15)	Test value (χ^2^)	p-value	Effect size, Cramer’s V
Pathological root resorption, n (%)	4 (26.7)	1 (6.7)	1 (6.7)	7.78	0.020*	0.416
Persistent or new periapical/furcation radiolucency, n (%)	5 (33.3)	2 (13.3)	1 (6.7)	7.03	0.030*	0.395

Predictors of overall treatment success

After adjusting for age, tooth type, arch, preoperative radiolucency, and obturation quality, the type of obturation material remained independently associated with overall treatment success. Compared with ZOE, Metapex was associated with greater odds of overall success (OR = 4.00, 95% CI: 1.08-14.81; p = 0.038), while Endoflas demonstrated an even stronger association (OR = 6.00, 95% CI: 1.48-24.30; p = 0.012). Optimal obturation was also independently associated with treatment success (OR = 3.00, 95% CI: 1.18-7.62; p = 0.023). By contrast, age, tooth type, arch, and preoperative radiolucency were not significant independent predictors of overall success. The regression model demonstrated good calibration according to the Hosmer-Lemeshow test (χ^2^ = 5.12, df = 8, p = 0.744), with a Nagelkerke R^2^ of 0.312 (Table 5).

**Table 5:** Binary logistic regression for factors associated with overall success at 12 months. Binary logistic regression was used to identify independent predictors of overall treatment success, with ZOE as the reference category for obturation material. The model was adjusted for age, tooth type, arch, preoperative radiolucency, and obturation quality; model calibration was assessed using the Hosmer-Lemeshow goodness-of-fit test, and Nagelkerke R^2^ was used to describe the model's explanatory capacity; *p < 0.05 was considered statistically significant. ZOE: zinc oxide eugenol; CI: confidence interval; ref: reference

Predictor	β coefficient	Adjusted odds ratio (OR)	95% CI for OR	Wald χ^2^	p-value
Obturation material (reference = ZOE)	-	-	-	-	-
Metapex (Group II)	1.386	4.00	1.08-14.81	4.32	0.038*
Endoflas (Group III)	1.792	6.00	1.48-24.30	6.25	0.012*
Age (per one‑year increase)	-0.095	0.91	0.65-1.28	0.28	0.597
Tooth type (molar, ref = first primary molar)	-0.223	0.80	0.41-1.56	0.42	0.515
Arch (mandibular, ref = maxillary)	0.405	1.50	0.65-3.46	0.91	0.340
Preoperative radiolucency (present, ref = absent)	-0.799	0.45	0.18-1.10	3.09	0.079
Optimal obturation (ref = non‑optimal)	1.099	3.00	1.18-7.62	5.15	0.023*

## Discussion

The present study demonstrated that the clinical and radiographic performance of the evaluated root canal filling materials were not equivalent. Calcium hydroxide-iodoform-based materials, particularly Endoflas, showed more favorable overall outcomes than ZOE. This finding was consistent across the composite clinical endpoint, radiographic healing, obturation quality, and occurrence of adverse radiographic changes. Importantly, the association between material selection and treatment success remained significant after adjusting for relevant clinical and radiographic variables, suggesting that the observed differences were not entirely explained by baseline characteristics.

The favorable performance of Endoflas may be explained by the combination of components with complementary properties. Calcium hydroxide provides a highly alkaline environment with antimicrobial potential, whereas iodoform contributes to antimicrobial activity and radiopacity [12]. The material is also designed to undergo resorption, an important characteristic of primary teeth, because the filling material should ideally remain compatible with physiological root resorption [12,13]. A systematic review specifically evaluating Endoflas reported favorable clinical and radiographic outcomes and highlighted its ability to reduce interradicular radiolucency and undergo relatively rapid resorption when extruded [12]. A more recent systematic review and meta-analysis comparing Endoflas with ZOE similarly concluded that Endoflas were associated with a lower risk of clinical and radiographic failure, supporting the direction of the present findings [14].

The better outcomes observed with Metapex than with ZOE may be related to its calcium hydroxide-iodoform formulation. The antimicrobial properties of these components may contribute to improved control of residual microorganisms following chemomechanical preparation [5,15]. In addition, the relatively favorable resorption characteristics of calcium hydroxide-iodoform pastes are particularly relevant when treating primary teeth approaching physiological exfoliation. However, the previous evidence is not entirely consistent. A systematic review and meta-analysis comparing calcium hydroxide-iodoform pastes with ZOE found no significant difference in clinical or radiographic success at shorter follow-up intervals, although differences emerged with longer observation [5]. Consequently, the authors emphasized that material selection should also consider the expected time for exfoliation. Estrela et al. [16] demonstrated that calcium hydroxide preparations containing iodoform exhibit antimicrobial effectiveness against several microorganisms, supporting the potential contribution of this combination to microbial control within the root canal system.

The quality of obturation is another important finding of the present study. Optimal filling was significantly more frequent with EndoFlas and Metapex than with ZOE. This may have directly contributed to their superior treatment outcomes because adequate obturation reduces the likelihood of persistent microbial contamination and provides a more effective seal of the prepared canal system [5,12,13]. The importance of filling quality is supported by recent evidence identifying adequate root canal filling as one of the factors consistently associated with successful pulpectomy outcomes [3]. Furthermore, the present regression analysis demonstrated that optimal obturation was independently associated with treatment success after adjustment for other variables. This reinforces the concept that the clinical performance of a material cannot be considered independently of the quality with which it is placed.

The difference in obturation quality observed in the present study may also be related to the physical handling characteristics of the filling materials and obturation techniques. Almutairi et al. [17] evaluated ZOE and Metapex using different obturation techniques in primary canines and found that obturation length and density varied according to both the filling material and technique. Their findings indicate that the quality of the final root canal filling is influenced not only by the material itself but also by its method of placement. This may partly explain the greater frequency of optimal obturation observed with Metapex and Endoflas in the present clinical study.

The lower performance of ZOE in the present study was particularly evident in the radiographic outcomes. Pathological root resorption and persistent or newly developed periapical/furcation radiolucency were more frequent with ZOE than with calcium hydroxide-iodoform-based materials. Singh et al. [18] reported similar results; they noticed a lower success rate with ZOE than with Endoflas. One possible explanation is the slower resorption of ZOE compared to the physiological resorption of primary roots, particularly when the material extends beyond the root apex [11]. A previous systematic review has similarly raised concerns regarding the persistence of extruded ZOE particles and the differences between the resorption behavior of ZOE and calcium hydroxide-iodoform formulations [19]. Nevertheless, ZOE remains a clinically established material, and its historical use and acceptable success rates indicate that its low performance in the present study should not be interpreted as evidence that it is unsuitable for all primary teeth.

The findings should also be interpreted in the context of conflicting comparative clinical evidence. A recent randomized clinical trial comparing Endoflas, Metapex, and another calcium hydroxide-based formulation reported comparable short-term clinical outcomes among groups [20]. These differences from the present findings may reflect variations in treatment protocols, follow-up duration, outcome definitions, case selection, obturation technique, and study design. In particular, the present study used a stringent composite endpoint requiring both clinical and radiographic success, which may distinguish materials more clearly than a clinical assessment alone. However, the broader evidence base remains unclear. A 2026 network meta-analysis concluded that Endoflas and ZOE-based formulations remain viable options but emphasized the low to very low certainty of much of the available evidence [21]. Therefore, although the present findings favor Endoflas and Metapex, they should be interpreted within the context of the continuing methodological limitations of the pediatric endodontic literature.

The quality of obturation is another important finding of the present study. Optimal filling was more frequently observed with Endoflas and Metapex than with ZOE. However, obturation quality should not be attributed solely to the intrinsic properties of the filling material. Canal anatomy, obturation technique, material handling characteristics, and the interaction between the sealer and filling material can influence the formation and distribution of voids. Experimental micro-computed tomographic studies have demonstrated differences in void volume according to obturation technique and sealer, while also showing that the proportion of voids may change substantially over prolonged periods after obturation [22,23]. Thus, radiographic or volumetric evidence of voids at a particular time point does not necessarily represent a fixed or direct measure of clinical failure. In particular, the clinical significance of a void may depend on its location and whether it constitutes a continuous pathway capable of facilitating microbial leakage.

In the present study, optimal obturation was independently associated with overall treatment success after adjustment for age, tooth type, arch, preoperative radiolucency, and obturation material. However, given the retrospective observational design, this association should not be interpreted as evidence that better obturation quality directly caused improved clinical or radiographic outcomes. Rather, adequate obturation may represent one component of successful treatment, potentially reflecting the combined influence of material properties, canal anatomy, obturation technique, and operator-related factors. Therefore, the present findings support an association between obturation quality and treatment outcome rather than a direct causal effect.

Clinical implications and limitations

From a clinical perspective, these findings support the consideration of calcium hydroxide-iodoform-based materials, particularly Endoflas, as alternatives to conventional ZOE when treating primary molars requiring pulpectomy. However, material selection should be individualized according to the extent of preoperative pathology, expected time to exfoliation, root resorption status, and the clinician's ability to achieve adequate obturation. The importance of obtaining optimal root canal filling should be emphasized, irrespective of the material selected.

The study was limited by its retrospective design, single-center setting, relatively small sample size, reliance on existing clinical and radiographic documentation, and potential selection and information biases. Material allocation was determined by routine clinical practice rather than randomization, leaving the possibility of residual confounding despite multivariate adjustment. The follow-up period was also relatively limited for evaluating the long-term behavior of the filling materials in the primary teeth. Larger multicenter prospective studies with standardized treatment protocols, blinded outcome assessment, and longer follow-up are warranted to establish the comparative effectiveness and long-term biological behavior of these materials.

## Conclusions

Within the limitations of this retrospective observational study, calcium hydroxide-iodoform-based materials, particularly Endoflas, were associated with more favorable clinical and radiographic outcomes than ZOE in primary molars. Better obturation quality is associated with treatment success. These findings suggest potential advantages of these materials in routine pediatric endodontic practice; however, they should be interpreted as associations rather than causal effects. Prospective multicenter studies with standardized treatment protocols and longer follow-up periods are warranted to confirm these observations.

## References

[1] Smaïl-Faugeron V, Glenny AM, Courson F, Durieux P, Muller-Bolla M, Fron Chabouis H (2018). Pulp treatment for extensive decay in primary teeth. Cochrane Database Syst Rev.

[2] Ramakrishnan M, Banu S, Ningthoujam S, Samuel VA (2019). Evaluation of knowledge and attitude of parents about the importance of maintaining primary dentition - a cross-sectional study. J Family Med Prim Care.

[3] Kendre SB, Bhatane AU, Kale YJ, Dadpe MV, Dahake PT (2025). Factors affecting success and failure of pulpectomy in primary teeth: a systematic review. Int J Clin Pediatr Dent.

[4] Navit S, Jaiswal N, Khan SA (2016). Antimicrobial efficacy of contemporary obturating materials used in primary teeth - an in-vitro study. J Clin Diagn Res.

[5] Najjar RS, Alamoudi NM, El-Housseiny AA, Al Tuwirqi AA, Sabbagh HJ (2019). A comparison of calcium hydroxide/iodoform paste and zinc oxide eugenol as root filling materials for pulpectomy in primary teeth: a systematic review and meta-analysis. Clin Exp Dent Res.

[6] Holan G, Fuks AB (1993). A comparison of pulpectomies using ZOE and KRI paste in primary molars: a retrospective study. Pediatr Dent.

[7] Spedding RH (1985). Incomplete resorption of resorbable zinc oxide root canal fillings in primary teeth: report of two cases. ASDC J Dent Child.

[8] Jara M, Salcedo-Moncada D, Ayala G, Watanabe R, Alvítez-Temoche D, Mayta-Tovalino F (2020). Antibacterial efficacy of calcium hydroxide with iodoform versus calcium hydroxide with camphorated paramonochlorophenol as intrachannel pastes on an Enterococcus faecalis biofilm: a comparative in vitro study. J Int Soc Prev Community Dent.

[9] Silva Junior MF, Wambier LM, Gevert MV, Chibinski AC (2022). Effectiveness of iodoform-based filling materials in root canal treatment of deciduous teeth: a systematic review and meta-analysis. Biomater Investig Dent.

[10] von Elm E, Altman DG, Egger M, Pocock SJ, Gøtzsche PC, Vandenbroucke JP (2008). The Strengthening the Reporting of Observational Studies in Epidemiology (STROBE) statement: guidelines for reporting observational studies. J Clin Epidemiol.

[11] Al-Ostwani AO, Al-Monaqel BM, Al-Tinawi MK (2016). A clinical and radiographic study of four different root canal fillings in primary molars. J Indian Soc Pedod Prev Dent.

[12] Elicherla SR, Bandi S, Venkata Saikiran K, Nunna M, Sai Sahiti P, Nuvvula S (2022). The clinical and radiographic success of Endoflas compared with other root canal obturating materials in primary teeth: a systematic review. Dent Res J (Isfahan).

[13] Pandranki J, V Vanga NR, Chandrabhatla SK (2018). Zinc oxide eugenol and Endoflas pulpectomy in primary molars: 24-month clinical and radiographic evaluation. J Indian Soc Pedod Prev Dent.

[14] Awad R, Musa M, Elhoumed M, Liu F, Guo Q (2024). Comparison of endoflas and zinc oxide eugenol as root canal filling materials for pulpectomy in deciduous teeth: a systematic review and meta-analysis. Saudi Dent J.

[15] Cwikla SJ, Bélanger M, Giguère S, Progulske-Fox A, Vertucci FJ (2005). Dentinal tubule disinfection using three calcium hydroxide formulations. J Endod.

[16] Estrela C, Estrela CR, Hollanda AC, Decurcio Dde A, Pécora JD (2006). Influence of iodoform on antimicrobial potential of calcium hydroxide. J Appl Oral Sci.

[17] Almutairi M, Almutairi R, Almogbel S, Alfarhan A, Alharbi W, Alayoub RA (2024). Comparative evaluation of the quality of obturation between three obturation techniques in primary canines: an in vitro study. Cureus.

[18] Singh S, Saxena A, Khandelwal V, Verma N, Jain A (2025). Evaluation of zinc oxide-eugenol, zinc oxide-propolis and endoflas as an obturating materials in primary molar pulpectomies - a comparative study. Indian J Dent Res.

[19] Rhaiem M, Elelmi Y, Baaziz A, Chatti M, Maatouk F, Ghedira H (2023). Alternative root canal filling materials to zinc oxide eugenol in primary teeth: a systematic review of the literature. Eur Arch Paediatr Dent.

[20] Khadilkar AS, Kapur A, Goyal A, Gauba K, Singh SK (2024). Comparison of clinical performance of obturating materials in pulpectomies: a randomized clinical trial. J Indian Soc Pedod Prev Dent.

[21] Patnana AK, Ali AR, Narayanan D, Dammuilung P, Sharma S, Kumar P (2026). Effectiveness of different obturation materials in primary teeth: a network meta-analysis. J Indian Soc Pedod Prev Dent.

[22] Liu H, Lai WW, Hieawy A (2021). Micro-computed tomographic evaluation of the quality of root canal fillings in mandibular molars after obturation for 54 months. J Endod.

[23] Zhang W, Liu H, Wang Z, Haapasalo M, Jiang Q, Shen Y (2022). Long-term porosity and retreatability of oval-shaped canals obturated using two different methods with a novel tricalcium silicate sealer. Clin Oral Investig.

